# Effects of chronic cobalt and chromium exposure after metal‐on‐metal hip resurfacing: An epigenome‐wide association pilot study

**DOI:** 10.1002/jor.23525

**Published:** 2017-02-09

**Authors:** Julia Steinberg, Karan M. Shah, Alison Gartland, Eleftheria Zeggini, Jeremy Mark Wilkinson

**Affiliations:** ^1^ Wellcome Trust Sanger Institute Cambridge UK; ^2^ Department of Oncology and Metabolism, The Mellanby Centre for Bone Research The University of Sheffield Sheffield UK

**Keywords:** metal‐on‐metal prostheses, cobalt, chromium, DNA methylation

## Abstract

Metal‐on‐metal (MOM) hip resurfacing has recently been a popular prosthesis choice for the treatment of symptomatic arthritis, but results in the release of cobalt and chromium ions into the circulation that can be associated with adverse clinical effects. The mechanism underlying these effects remains unclear. While chromosomal aneuploidy and translocations are associated with this exposure, the presence of subtle structural epigenetic modifications in patients with MOM joint replacements remains unexplored. Consequently, we analyzed whole blood DNA methylation in 34 OA patients with MOM hip resurfacing (MOM HR) compared to 34 OA patients with non‐MOM total hip replacements (non‐MOM THR), using the genome‐wide Illumina HumanMethylation 450k BeadChip. No probes showed differential methylation significant at 5% false‐discovery rate (FDR). We also tested association of probe methylation levels with blood chromium and cobalt levels directly; there were no significant associations at 5% FDR. Finally, we used the “epigenetic clock” to compare estimated to actual age at sample for all individuals. We found no significant difference between MOM HR and non‐MOM THR, and no correlation of age acceleration with blood metal levels. Our results suggest the absence of large methylation differences systemically following metal exposure, however, larger sample sizes will be required to identify potential small effects. Any DNA methylation changes that may occur in the local periprosthetic tissues remain to be elucidated. © 2017 The Authors. Orthopaedic Research Society. Published by Wiley Periodicals, Inc. on behalf of Orthopaedic Research Society. J Orthop Res 35:2323–2328, 2017.

Metal‐on‐metal (MOM) hip replacement and resurfacing have been, until recently, popular treatments of choice for patients with painful arthritis,^1^ with over 1.5 million devices inserted worldwide.^2^, ^3^ Wear and corrosion occur at the bearing surfaces of MOM prostheses and at modular junctions of all hip prostheses.^4^, ^5^ This causes the release of metallic wear debris into the local tissues and peripheral circulation, resulting in a systemic elevation of cobalt and chromium concentrations.^6^, ^7^

The adverse effects of elevated levels of metal exposure have been highlighted by the high failure rates of MOM prostheses,^8^, ^9^ and by case reports of cardiac, neurological, and endocrine disorders,^10^ resulting in their recent recall from many markets.^11^ Several investigators have described the detrimental effects of metal exposure on survival and function of cell types including bone cells, monocytes, and macrophages in vitro.^12^, ^13^, ^14^, ^15^ The mechanism by which metal debris exerts these effects is still unclear, although some investigators have studied changes in gene expression of candidate genes in cell and animal models.^16^, ^17^, ^18^

Several studies have reported metal‐induced changes in DNA methylation, alteration in gene expression and cell physiology in the setting of cancer.^19^, ^20^, ^21^ In particular, chromium (Cr) induces oxidative stress, leads to gene expression changes associated with cell‐to‐matrix adhesion in cell lines, and affects the methylation of several cancer genes in cell lines.^20^ Furthermore, cobalt (Co) exposure leads to chromosomal breaks and aberrations in mice, and to DNA damage in human cell lines, likely due to inhibition of DNA repair.^22^ However, the effects of chronic metal exposure on the patient epigenome and cell function in the setting of joint replacement remain unexplored.

Here, we tested whether DNA methylation levels of over 400,000 loci differ between whole blood samples from MOM hip resurfacing patients (MOM HR) and conventional total hip replacement using a non‐MOM bearing (non‐MOM THR). We also directly tested the association of methylation levels with circulating metal levels.

## METHODS

### Participants and Phenotypes

Patients were recruited from the community between November 24th, 2009 and May 20th, 2010, and provided written informed consent prior to participation. All patients were recruited from the operating records of a single surgeon, and the study was approved by the National Research Ethics Service (South Yorkshire REC 09/H131/62 and South Central REC 10/H0606/20). All methods were performed in accordance with the relevant guidelines and regulations.

Patients with MOM HR that present with a pathology due to elevated circulating metal concentrations, subsequently undergo a revision surgery that eventually reduces the concentrations of metal in circulation.^23^, ^24^ It is therefore the patient population with a well‐functioning MOM hip replacement which have low‐level chronic exposure to circulating metals, for whom the longer term outcomes are unclear. The exposure cohort in the study consisted of a metal exposure group of 35 patients with a well‐functioning MOM HR. These were individually matched for gender to a non‐exposure group consisting of patients who had a received non‐MOM THR, and were of similar age and time‐since‐surgery (Table 1). Whole blood samples were collected from all patients and Co and Cr levels measured by inductively‐coupled plasma‐mass spectroscopy (ICP‐MS), as described previously^25^ (Table 1). Undetectable levels of Cr and Co were imputed as half the lowest observed value for that phenotype. Smoking was measured as a binary variable, indicating whether an individual had ever smoked (self‐reported). Occupational and other non‐arthroplasty environmental metal exposure was measured as a self‐reported binary variable.

**Table 1:** Patient Characteristics and Metal Levels for Patients With Metal‐on‐Metal Hip Resurfacing (MOM HR) and a Non Metal‐on‐Metal Total Hip Replacement (Non‐MOM THR)

	MOM HR	Non‐MOM THR
Gender		
Male	30	30
Female	4	4
Smoking (current or past)		
Yes	12	12
No	22	22
Non‐TJR metal exposure		
Yes	4	12
No	30	22
Age at sample (years)		
Median (range)	59.7 (49.2–75.7)	59.75 (47.5–75.3)
Time since operation (years)		
Median (range)	9.75 (4.9–12.1)	8.75 (1.8–15.9)
Cobalt (μg/L)		
Median (range)	1.84 (0.56–36.99)	0.60 (0.17–4.52)
Chromium (μg/L)		
Median (range)	2.52 (0.44–27.54)	0.34 (0.12–3.01)

Co and Cr levels showed high correlation (*r* = 0.95, *p *< 10^−10^). As expected, Co and Cr metal levels were significantly higher in 34 exposed than in 34 non‐exposed subjects who passed quality control (Wilcox *p *< 10^−9^; Table 1).

### DNA Extraction

Genomic DNA was isolated from the whole blood samples using the QIAamp DNA Blood Midi Kit (Qiagen, Hilden, Germany) according to manufacturer's protocol.

### Methylation: Illumina 450k BeadChip Assay

The DNA samples were quantified using NanoDrop 2000 and A_260/280_ ratio was used to assess for the presence of protein contaminants. The DNA sample concentrations were then normalized to 50 ng/μl. Bisulfite conversion of DNA was done using the Zymo EZ‐96 DNA Methylation Assay as per manufacturer's guidelines (Zymo Research Corp., Irvine, CA). The DNA methylation levels in the bisulfite converted DNA was measured using the Illumina Infinium HD Methylation Assay (Illumina Inc, San Diego, CA) according to the manufacturer's instructions. Briefly, bisulfite DNA was isothermally amplified (20–24 h) and fragmented enzymatically. Subsequently, the DNA was precipitated using isopropanol, resuspended in a hybridization buffer, and dispensed onto Illumina Infinium HumanMethylation 450k BeadChips (12 samples/chip) for hybridization at 48°C (16–20 h) in the Illumina Hybridisation Oven. The unbound and non‐specifically bound DNA fragments were washed away and a single nucleotide extension of the BeadChip bound fragments was performed to incorporate the fluorescent (ddNTP) labels. The entire protocol was automated using the Freedom Evos robot (Tecan). The BeadChips were imaged using an Illumina iScan.

### Methylation Data Processing

Initial quality control of samples was performed using Illumina GenomeStudio software by comparing the sample probe signal intensities with inbuilt control probes. Subsequently, the probes were filtered using the *ChAMP* package in R.^26^ The un‐hybridized probes (detection *p*‐value > 0.01; *n* = 2,265), probes mapping to the sex chromosomes or those containing common SNPs within 2 base‐pairs of the CpG site (based on minor allele frequency of over 5% in 1000 Genomes Project Consortium 2010 data) were excluded. Additionally, probes that were not represented by a minimum of three beads in 5% of samples (*n* = 549) and those that cross‐hybridize with multiple genomic location were also excluded.^27^ Following the filtering process, a total of 426,225 probes were taken forward for subsequent analysis. All individuals had probe failure rates below 1%. Normalization procedures to correct for background differences, dye‐bias and differences in the probe chemistries were conducted using the “*Dasen*” pre‐processing method in the *wateRmelon* R package.^28^

### Estimation of Cell Composition

Since methylation levels were assessed in whole blood, the heterogeneity in the cell type composition between patients could influence the results and needs to be accounted for.^29^ Cell composition for each individual were estimated using the function “*estimateCellCounts*” in the R package *minfi*.^30^ This function infers the proportions of CD8+ T‐cells, CD4+ T‐cells, B cells, natural killer (NK) cells, monocytes, and granulocytes for each sample, based on DNA methylation signatures of different the cell types.^29^ One MOM HR sample had cell type proportion estimates outside of six median absolute deviations from the median of all samples; this sample and the corresponding matched non‐MOM THR sample were excluded from all further analyses. We checked that all remaining samples had cell‐type composition estimates consistent with ranges observed in large‐scale studies of individuals of similar age.^29^ Thirty‐four individuals with MOM HR and 34 individuals with non‐MOM THR were taken forward for further analyses (Table 1).

### Sample Clustering

Previous studies investigating genome‐wide DNA methylation in OA patients have reported clustering of samples based on the global similarities of their methylation patterns.^31^, ^32^ The samples were assessed for clustering using an unsupervized hierarchical clustering algorithm that forms groups by successively merging samples based on the similarity of their methylation patterns. The analyses were performed by using the “*hclust*” function in R. We accounted for sample gender, age‐at‐sample, time‐since‐operation, smoking, metal exposure due to other causes, such as via any occupational metal exposure or via hobbies, and cell composition by using residuals from linear regression of beta‐values on these covariates. To confirm the clustering results, we also carried out a principal component analysis (PCA) on the residuals of beta values as used in the clustering, applying the “*prcomp*” function in R.

### Analysis of Differentially Methylated Probes (DMPs)

After quality control, we analyzed 426,225 CpG sites in 34 MOM HR and 34 non‐MOM THR samples. To identify DMPs between the MOM and non‐MOM samples, a logistic regression model for each CpG site was fitted using the *CpGassoc* R package,^33^ adjusting for the covariates of gender, age‐at‐sample, time‐since‐surgery, smoking status, non‐hip replacement metal exposure, and cell composition. Similar association analyses were also performed for plasma Cr and blood Co levels: In each case, the Cr or Co level was log 10‐transformed, and a linear regression model was fitted with the same ten covariates as above. The analyses were also repeated with the first PC as an additional covariate. DMPs in a given analysis were defined as probes with a difference significant at 5% False Discovery Rate (FDR).

### Analysis of Methylation Age

Methylation age (DNAmAge) was analyzed using the online “DNA Methylation Age Calculator” (https://dnamage.genetics.ucla.edu/, accessed 17/09/2015), with the pre‐QC non‐normalized data as recommended (https://labs.genetics.ucla.edu/horvath/dnamage/faq.htm, last accessed 11/02/2016). This method is based on methylation levels at 353 CpG sites that strongly correlate with chronological age of across several tissues.^34^ Age acceleration was measured as residuals of DNAmAge regressed on age‐at‐sample, as recommended. Differences in age acceleration between MOM HR and non‐MOM THR samples were analyzed using the “*wilcox.test*” function in R, and correlation with circulating metal levels was tested using the “*cor.test*” function with *method* *= *“*spearman*” in R.

### Statistical Analyses

All analyses were performed in R version 3.1.2, with packages and functions as listed above.

## RESULTS

### Estimation of Cell‐Type Composition

**Figure 1:**
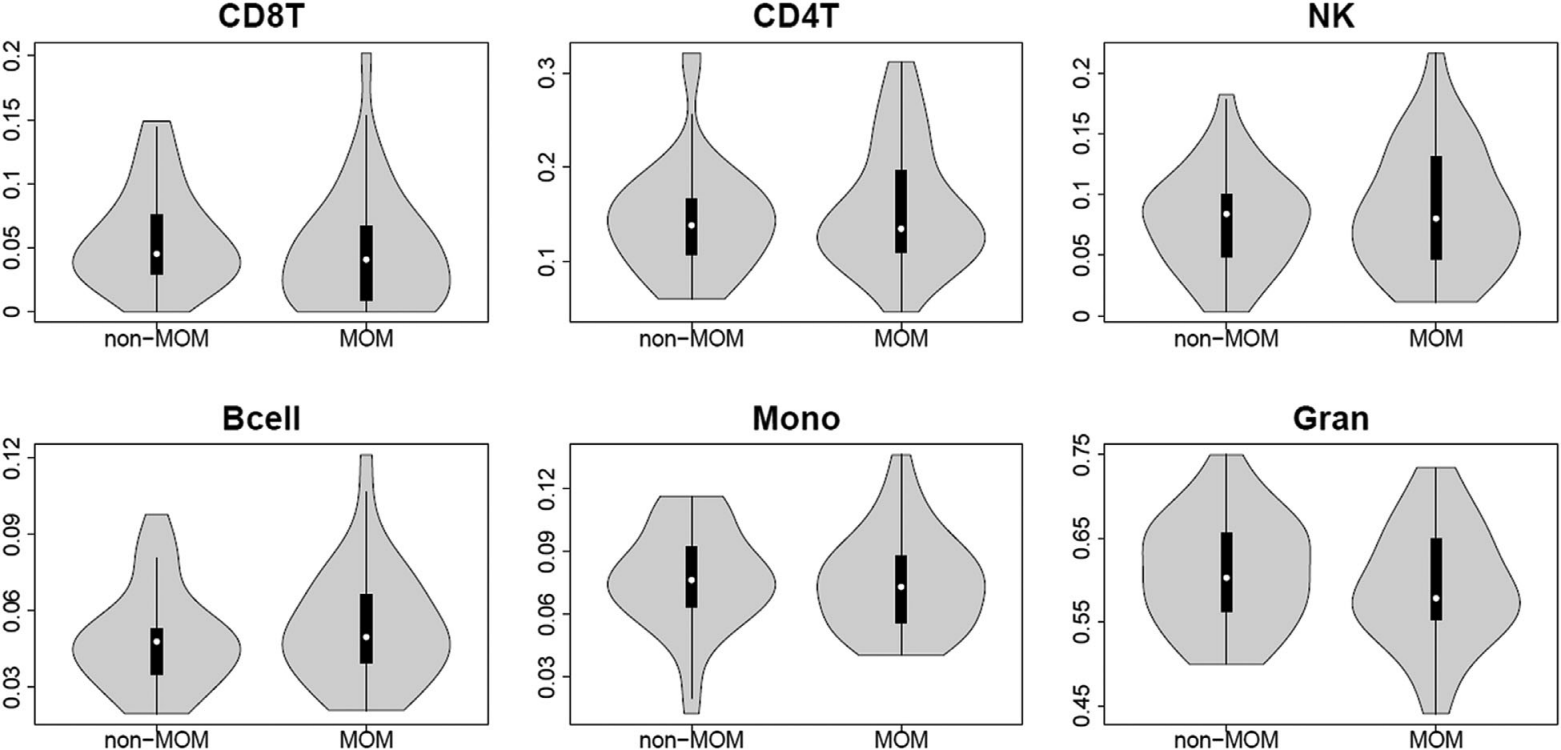
Cell‐composition estimates. Violin plots of different cell‐type (CD8+ T‐cells, CD4+ T‐cells, natural killer (NK) cells, B‐cells, monocytes and granulocytes) illustrating the distributions for non‐MOM THR and MOM HR samples after quality control. The white point shows the median value.

Since methylation levels were assessed in whole blood and thus could be influenced by cell type heterogeneity, we estimated the cell type composition of all samples.^35^ There were no significant differences in estimated proportions of CD8+ T‐cells, CD4+ T‐cells, B cells, natural killer (NK) cells, monocytes, and granulocytes between MOM HR and non‐MOM THR samples (Wilcoxon test *p*‐value > 0.17 for difference in each of the cell types; Fig. 1). Nonetheless, we used the cell type composition estimates as covariates in further analyses.

### Sample Clustering

**Figure 2:**
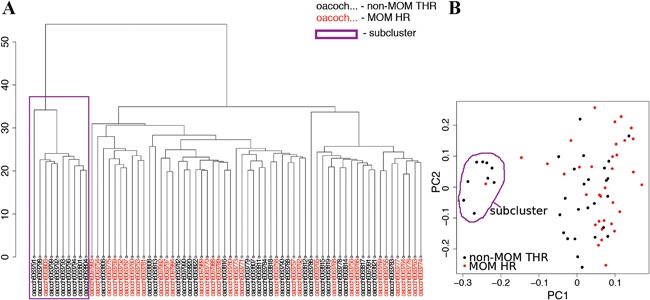
Sample clustering. (A) A dendrogram depicting results of unsupervized clustering using the “*hclust*” function in R shows a formation of a sub‐cluster (purple box), (B) which is confirmed using principal component analysis. Samples forming the sub‐cluster are adjacent in the methylation plate and likely represent a technical issue.

A sub‐cluster of ten individuals (one MOM HR, nine non‐MOM THR) was identified using hierarchical clustering, and confirmed by Principal Component Analysis (PCA) (Fig. 2A and B). This clustering could be attributed to technical reasons as all ten samples were located on the same methylation BeadChip (each BeadChip contains 12 samples). Hence the first principal component was used in subsequent analyses to account for the clustering.

### Differentially Methylated Probes (DMPs)

**Figure 3:**
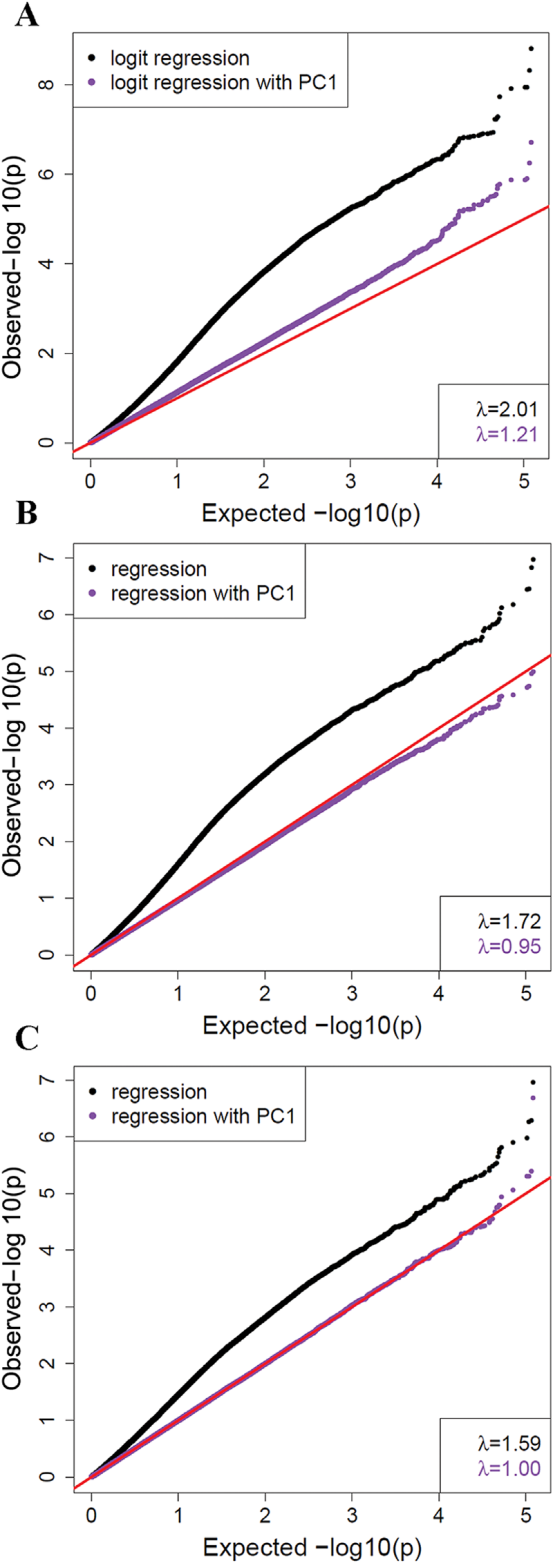
Quantile‐quantile plots for association of probe methylation levels with either (A) sample case/control status, (B) cobalt levels, or (C) chromium levels. The *x*‐axis is −log10 of the expected *p* values under the null hypothesis of no association, and the *y*‐axis is −log10 of the observed *p* values. Red line represents equality between observed and expected values. The *p* values were obtained from logistic regression using ten covariates (black), with a very high inflation (λ values inset) suggesting potential confounding. Logistic regression with an additional co‐variate of 1st principal component (PC, purple) to account for sub‐clustering of samples (potentially due to technical confounding) shows reduced inflation.

The probes were tested for differential methylation between MOM HR and non‐MOM THR samples, using gender, age at sample, time since surgery, smoking, metal exposure not due to MOM HR, estimated cell type proportions, and the first principal component as covariates. No probe was significantly associated at 5% FDR, with only 58 probes showing association at 25% FDR (Fig. 3A). When association of methylation levels with blood cobalt (Fig. 3B) and chromium levels (Fig. 3C) was tested, no probe showed a significant association with either of the metal levels at 5% FDR.

### Methylation Age

**Figure 4:**
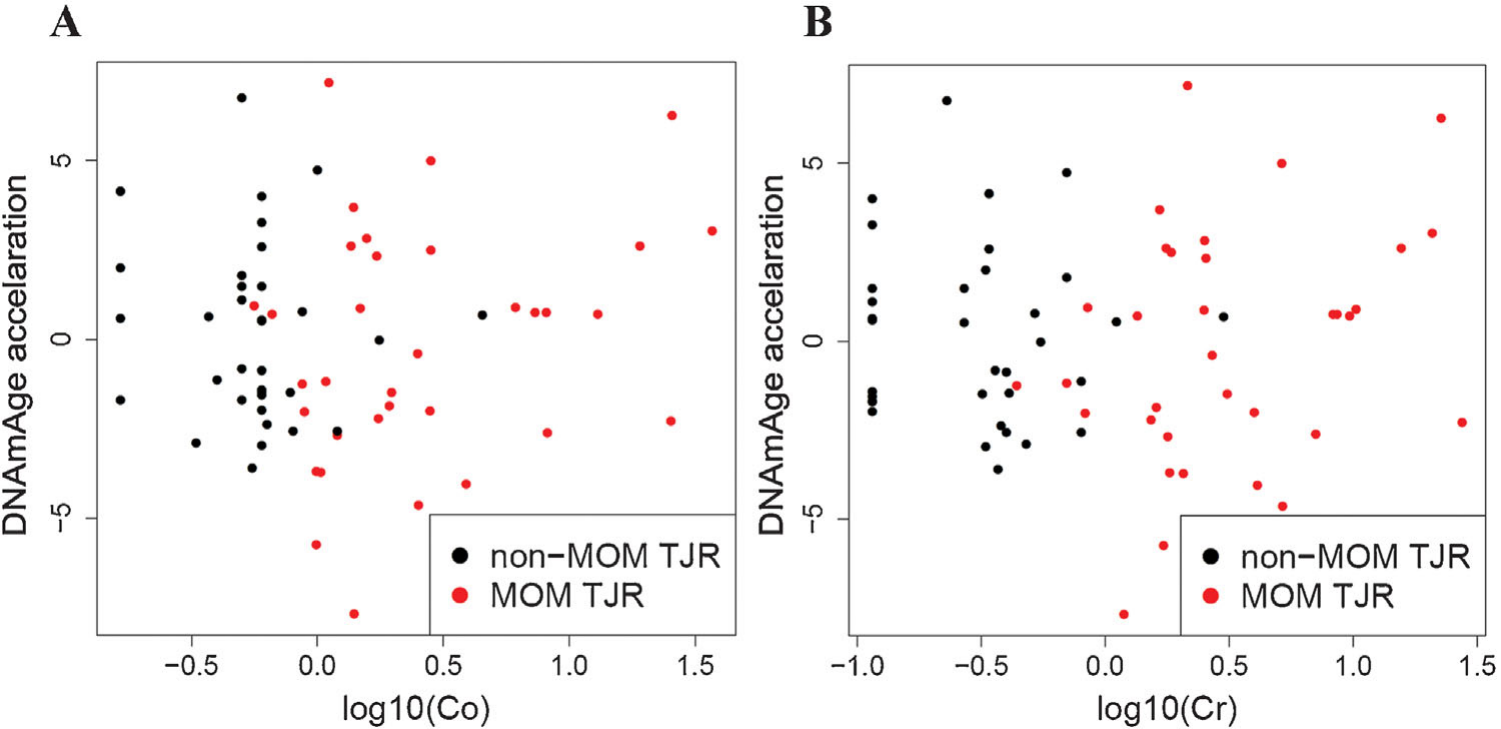
DNAmAge acceleration and (A) cobalt or (B) chromium levels in samples. The *y*‐axis is the measure of age acceleration (residuals from regression of DNAmAge on age‐at‐sample), the *x*‐axis shows the cobalt or chromium levels (log10‐transformed). There is no significant correlation of age acceleration with either cobalt or chromium levels (*p > *0.9).

In the 68 individuals that passed QC, age estimated from methylation (DNAmAge) was strongly correlated with actual age at sample (*r* = 0.89, *p *< 10^−10^). The age acceleration measure (residuals from regression of DNAmAge on age‐at‐sample) did not differ significantly between exposed and non‐exposed cases (Wilcox *p *> 0.7), and was not significantly correlated with plasma chromium or blood cobalt levels (*p > *0.9) (Fig. 4).

## DISCUSSION

Metal‐on‐metal hip resurfacing is known to increase circulating cobalt and chromium levels in blood, to reduce cell survival and function,^12^, ^13^, ^17^, ^18^ increase prosthesis failure,^8^, ^9^ and rarely, lead to systemic illness.^10^ To explore one potential mechanism for detrimental effects, we investigated blood DNA methylation differences between 34 osteoarthritis (OA) patients with metal‐on‐metal hip resurfacing (MOM HR), and 34 OA patients with a conventional total hip replacement (non‐MOM THR). To our knowledge, this is the first epigenome‐wide study comparing MOM HR to non‐MOM THR.

We did not find any significant methylation differences between MOM HR and non‐MOM THR samples, nor any significant methylation associations with either blood cobalt or chromium concentrations. Moreover, clustering and PCA analyses did not show a clear separation of MOM HR and non‐MOM THR samples, which suggests the absence of widespread global methylation differences.

We also estimated biological age of the samples as DNA methylation age, and used it to calculate age acceleration (residuals of the DNA methylation age regressed on actual age at sample). It has been shown that methylation data can be used to estimate the biological age of an individual.^34^ The predicted age can be used to measure “age acceleration,” that is, the difference between predicted and actual age. Age acceleration is highly heritable,^34^ significantly correlated with physical and cognitive fitness,^36^ and predicts all‐cause mortality later in life.^37^ We did not find any difference in age acceleration with MOM HR exposure, and no significant correlation of age acceleration with circulating blood metal levels.

The study was well‐powered to detect large methylation effects (∼100% power for differences over 25%), and the results indicate that such large effects are absent.^38^ The power for smaller effects dropped sharply (with <50% power to detect methylation differences of 20% at genome‐wide significance level), so that much larger samples will be needed to clarify the presence and extent of small methylation changes. For example, an estimated sample of 500 cases, 500 controls would be required to achieve over 80% power to detect a 5% case/control difference.^38^

In summary, our data suggest that systemic metal exposure at the level experienced by patients with well‐functioning metal‐on‐metal prostheses does not associate with large‐effect genomic DNA methylation differences in circulating cells when compared to age, sex, and time since surgery matched patients who had received a non‐MOM bearing total hip replacement. The changes that may occur in the local tissues surrounding the joint which are likely to be exposed to much higher levels of metal debris have not been examined in this study. Additionally, our study did not include patients with extreme levels of metal exposure, and large samples will be needed to clarify the presence of more subtle methylation changes than examined here.

## AUTHORS' CONTRIBUTIONS

JMW and AG designed the study. KS performed blood DNA extractions. JS designed and performed all computational analyses. All authors contributed to interpretation of the analyses. JS, KS, and JMW wrote the manuscript. JS, KS, JMW, EZ, and AG edited the manuscript. All authors read and approved the final manuscript.
